# Supplementary material for: Modeling the Electronic Absorption Spectra of the Indocarbocyanine Cy3
Source: Molecules. 2022 Jun 24;27(13):4062. doi: 10.3390/molecules27134062 (PMC9268038; doi:10.3390/molecules27134062)
Supplement: Supplementary file 1 [file molecules-27-04062-s001.zip › Supplementary-Material.pdf]

SUPPLEMENTARY MATERIAL

Modeling the Electronic Absorption Spectra of the Indocarbocyanine Cy3

Mohammed I. Sorour,*,† Andrew H. Marcus,*,‡ and Spiridoula Matsika*,†

†*Department of Chemistry, Temple University, Philadelphia, Pennsylvania 19122, United States*

‡*Department of Chemistry and Biochemistry, University of Oregon, Eugene, OR 97403, United States*

E-mail: Mohammed.Sorour@temple.edu; ahmarcus@uoregon.edu; smatsika@temple.edu

In this document we provide extra information on MD simulations, the Herzberg-Teller effect, and other effects. Coordinates of the ground and first excited state equilibrium structures obtained from B97XD/Def2SVP are also included.

1 Classical Ensemble: MD-QM/MM

1.1 MD Simulation and Equilibrium Criteria

The MD simulation was run for 200 ns using a Cy3-DNA construct adopted from the work of Heussman *et al.* [5] as shown in Figure S1b. The root mean square deviation (RMSD) and selected geometrical parameters are shown in Figure S2.

Cy3's dynamics was found to be dominated by π-stacking with the adjacent thymine on the same strand and the neighboring guanine from the opposite strand, see Figure S1b. The stabilization of Cy3 by π-stacking in its local environment is correlated to global stabilization of the whole Cy3-DNA construct. This can be shown by the global root mean square deviation (RMSD) analysis on the DNA which shows that the DNA reaches equilibrium around 150 ns, see Figure S2a.

Around the same time point (150 ns), Cy3 reached equilibrium within its local DNA environment driven by sandwich π-stacking with the neighboring thymine and guanine bases as shown in Figure S2b. The histograms in Figures S2c and S2d show the distribution of the configurations obtained from the MD after 150 ns as a function of the center of fragments separation between Cy3 and guanine and thymine, respectively. The neighboring thymine tends to be on average distance of 3.78 Å from Cy3, while the six-membered ring of guanine resides at distance around 4.2 Å.

1.2 Sampling criteria

Based on the structural analysis and equilibrium criteria shown in Figure S2, 301 uncorrelated configurations that span the equilibrium range (150-200 ns) were sampled in three

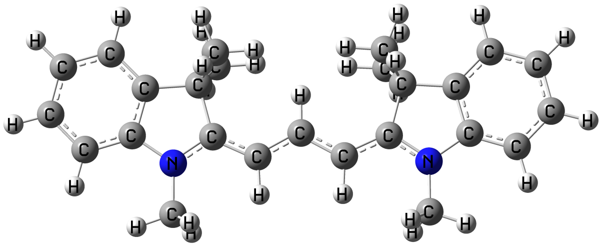

(a)

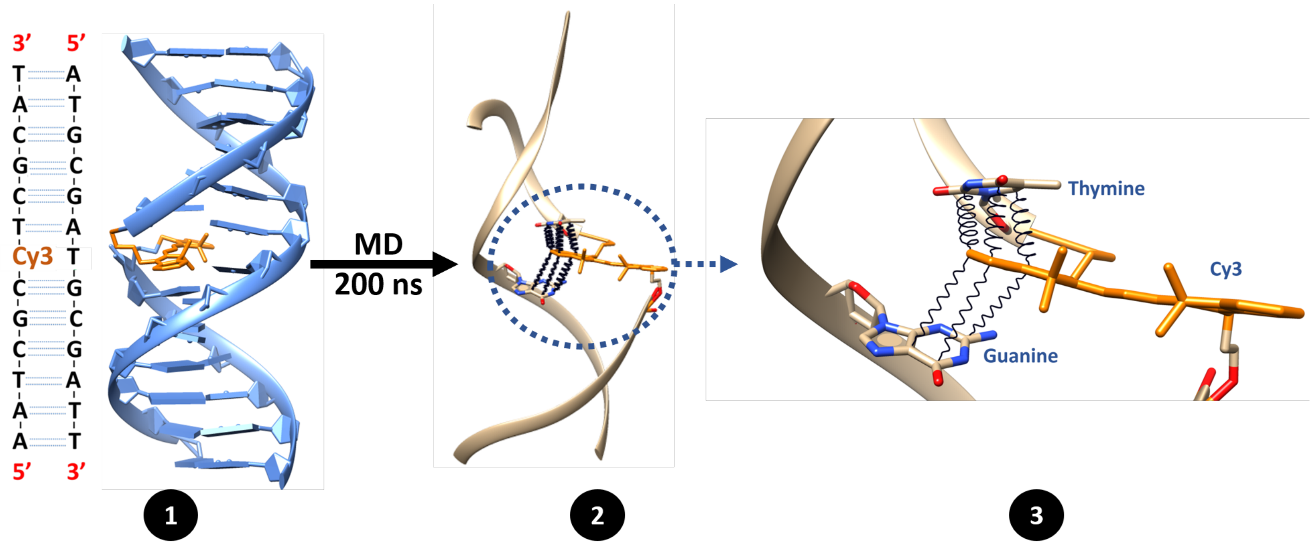

(b)

Figure S1: (a) Cy3 structure (b) Cy3-DNA sequence and structure analysis after approaching equilibrium. After equilibrium Cy3 structure is manifested by sandwich π-stacking between the thymine and guanine residues in its vicinity.

sets. The first set contains 101 frames starting from 150 ns and collected every 0.5 ns. The second and third sets contain 100 frame each, and the sampling started from 150.2 ns and 150.4 ns, respectively, and were collected in the same frequency as the first set.

1.3 QM/MM-MD: Electronic Structure Effect

As discussed in section 3.1.1, the electronic structure methods were found to have a prominent effect on the spectral shapes.

Figure S3 shows a comparison between the S_1 transition energies, transition dipole mo-

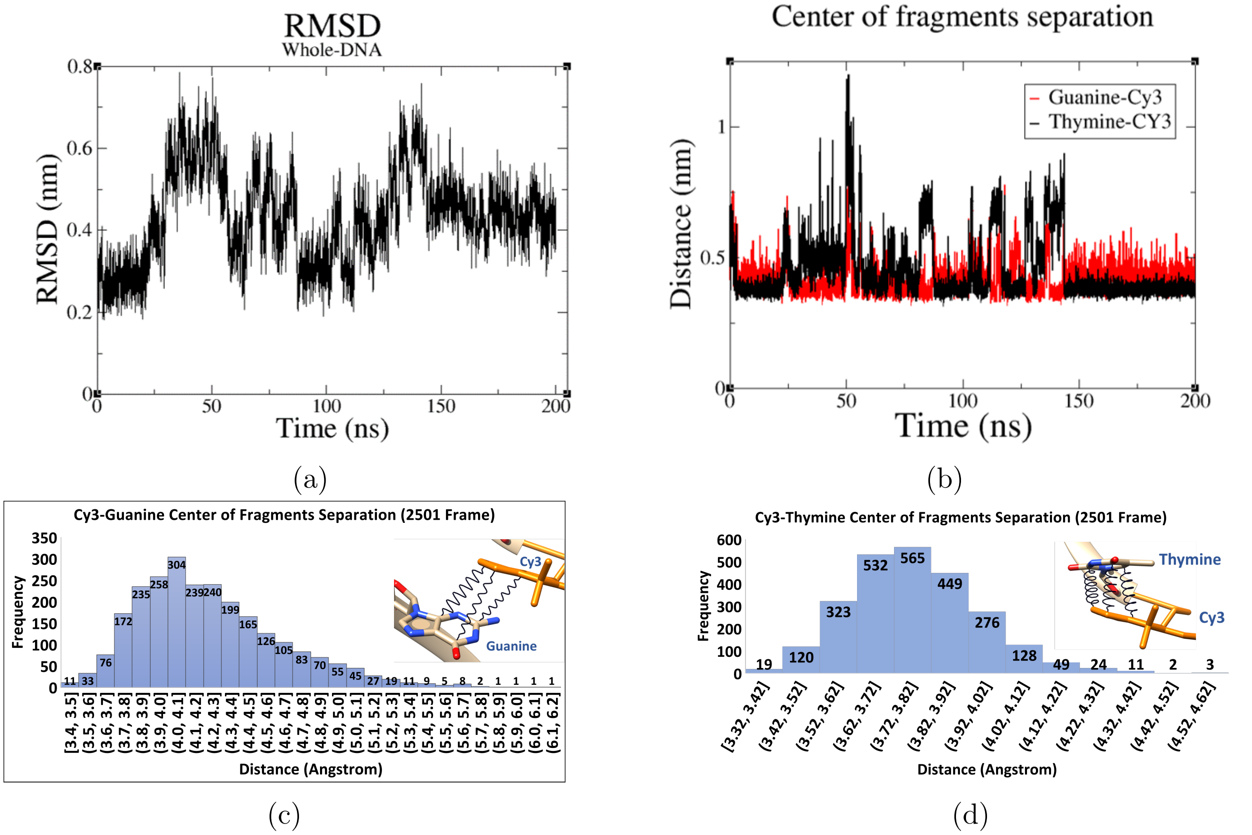

Figure S2: (a) Global DNA RMSD analysis. (b) Separation distance between one of the Cy3 six-membered rings and the six-membered rings of thymine and guanine as a function of the simulation time. (c) Distribution of the obtained frames at equilibrium as a function of the separation between guanine and Cy3. (d) Distribution of the obtained frames at equilibrium as a function of the separation between thymine and Cy3.

ments, and the oscillator strengths obtained from CC2 and ADC(2) calculations on top of 100 configurations. Figure S3a shows the S_1 transition energies differences in eV ($\Delta E/eV$) calculated as $E(S_1^{CC2}) - E(S_1^{ADC(2)})$; $\Delta E/eV$ fluctuates around an average value of 0.25 eV with a standard deviation of ∼0.05 eV. The fluctuations are more prominent for the transition dipole moment difference ($\Delta TDP/D$) which is calculated as $TDP(S_1^{CC2}) - TDP(S_1^{ADC(2)})$ which has a standard deviation ∼0.5 Debye, as shown in Figure S3b. These inconsistencies in the calculated transition energies and transition dipole moments explain the fluctuations observed for the oscillator strength, and as a consequence we obtain different spectral shapes.

In fact the inconsistencies in the shapes of the spectra obtained using CC2 and ADC(2)

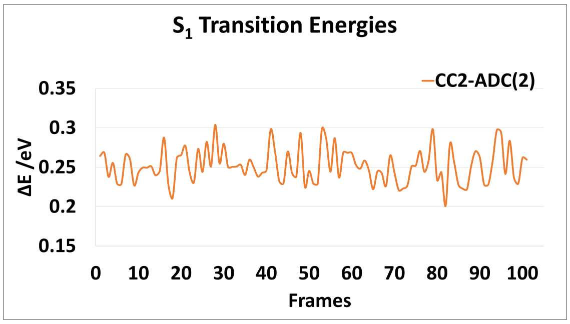

(a)

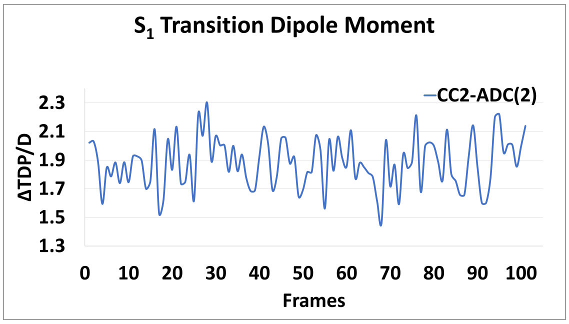

(b)

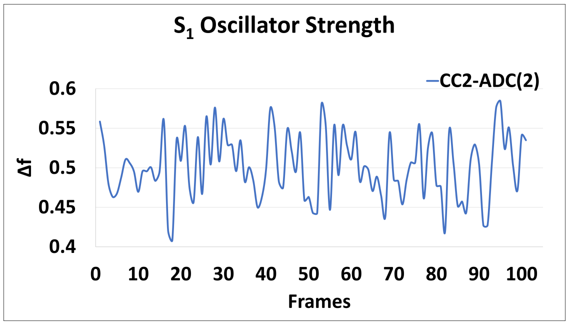

(c)

Figure S3: Comparing the transition energies and properties obtained from CC2 and ADC(2) over 100 frames. (a) First excited state excitation S_1 energy difference in eV ($\Delta E/eV$) calculated as $E(S_1^{CC2}) - E(S_1^{ADC(2)})$, (b) S_1 transition dipole difference in Debye ($\Delta TDP/D$) calculated as $TDP(S_1^{CC2}) - TDP(S_1^{ADC(2)})$, (c) S_1 oscillator strength (Δf) calculated as $f(S_1^{CC2}) - f(S_1^{ADC(2)})$.

are not as severe as CIS and CIS(D); the shape of the spectra obtained from CC2 and ADC(2) is similar, while those obtained from CIS and CIS(D) are quite different and that is mainly because of the fluctuation in the transition energies as they share the same transition dipole moment values. Figure S4 shows comparisons between the transition energies, transition dipole moments, and the oscillator strengths obtained from all the methods on the same configurations. Most of the TD-DFT methods have a consistent and a quite close performance among each other which explains the similar spectral shapes obtained from them.

It is worth mentioning that the CC2 QM/MM-MD calculation not only produces a spectrum that is closest to the correct experimental shape, but also it predicts the best energetics and transition dipole moment. The λ_{max} obtained from CC2 deviates from the experimental

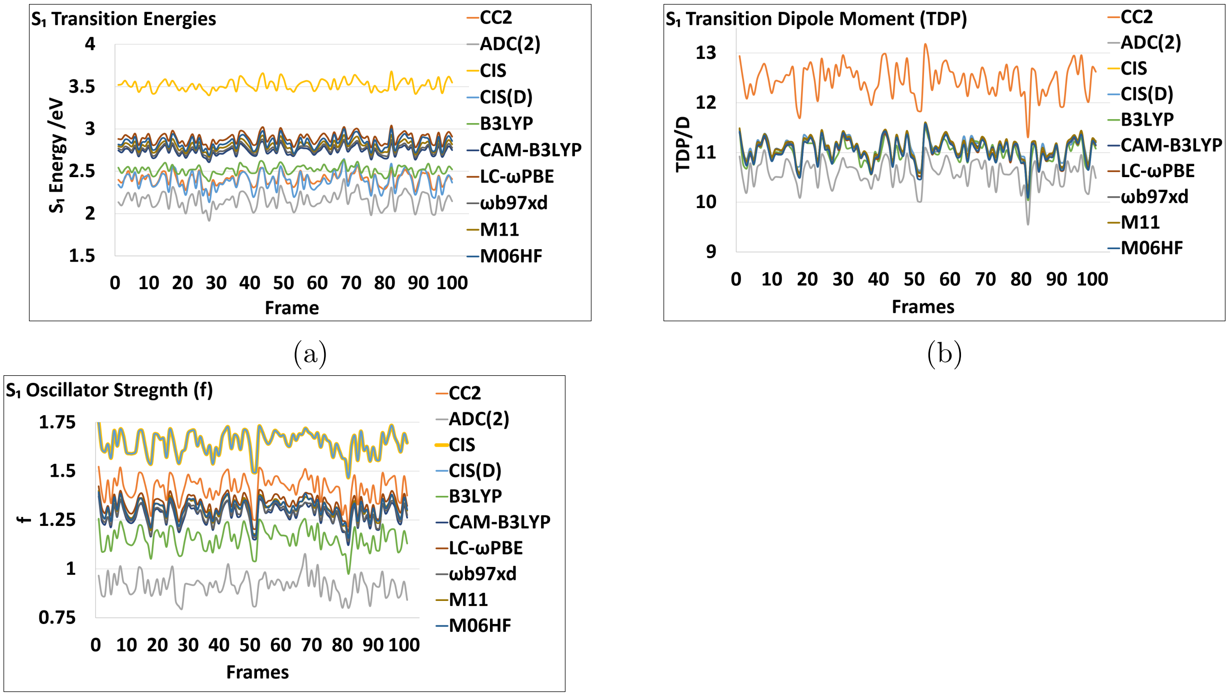

(a) (b)

(c)

Figure S4: Comparing the transition energies and properties obtained from all methods over 100 frames. (a) First excited state excitation S_1 energy in eV, (b) S_1 transition dipole (TDP) in Debye, (c) S_1 oscillator strength (f).

λ_{max} by 900 cm^{-1} (0.11 eV), while the average transition dipole moment is 12.46 Debye compared to an experimentally reported 12.8 Debye for the same system [5]. All the average values from all the methods are reported in Table S1.

Despite the apparent great performance of CC2, some of the results might be fortuitous. It is well established that the transition dipole moment is very sensitive to the formalism used for their derivations. The transition dipoles reported here are in the length gauge and they can differ by 7% from the velocity and mixed gauges for the same method. The inconsistencies of the transition dipole can be even more severe as it was reported before that the transition dipoles calculated from the linear response formalism can differ from the intermediate state representation by 0.8 D [75].

Lastly, we found a weak correlation between the spectral intensities obtained from the different methods in our QM/MM-MD simulations (Figure 2a) and the oscillator strength,

Table S1: Averages of the S_1 transition energies and properties obtained from all methods over 300 frames. TDP is the S_1 transition dipole in Debye, E(S_1) is the transition energy in eV, and f is the oscillator strength. The experimental E(S_1) and TDP are 2.56 eV and 12.8 Debye, respectively.

	CC2	ADC(2)	CIS	CIS(D)	B3LYP
E(S_1)	2.41	2.15	3.52	2.38	2.52
TDP	12.46	10.58	11.09	11.09	11.00
f	1.42	0.91	1.64	1.64	1.15

	CAM-B3LYP	ωB97XD	LC-ωPBE	M11	M06HF
E(S_1)	2.74	2.76	2.89	2.80	2.84
TDP	11.08	11.07	11.02	11.10	11.03
f	1.27	1.28	1.33	1.31	1.31

this is shown in Figure S5. This is expected since the intensity ε is calculated using the individual oscillator strengths. We found no correlation between the spectral intensities and the excitation energies and/or the transition dipole moment.

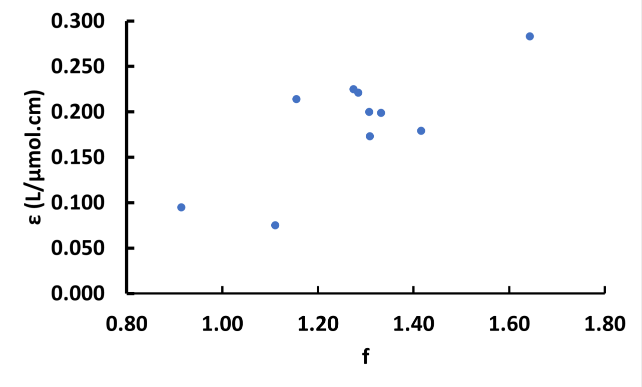

Figure S5: A correlation plot between the molar absorptivity ε in $L/\mu mol.cm$ at maximum absorption (Figure 2a) and the oscillator strength f.

1.4 Force Field Effect

As discussed in Section 3.1.3, the polymethine chain is thought to play an important role in the spectral signatures of Cy3. Table S2 shows a comparison between the AMBER-DYE [46] and CHARMM-DYE [86] force fields. The refined parameters obtained by Shaw *et al.*

[86] did not improve the bond parameters along the polymethine chain compared to those obtained by Graen *et al.* [46]. In fact, the parameters obtained by Shaw *et al.* [86] predicted a very small R(2,3) bond length of 1.19 Å which is almost the length of a C-C triple bond. The atom types listed in Table S2 correspond to the atom types in the original force fields [46,86].

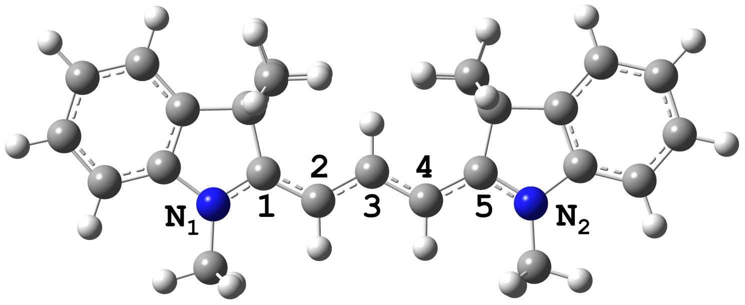

Figure S6: Cy3 structure optimized using ωB97XD/Def2SVP, and showing the numbering of the polymethine chain atoms.

Table S2: Comparison of the polymethine bond length obtained from AMBER-DYE [46] and CHARMM-DYE [86] force fields. The atom types listed in Table S2 correspond to the atom types in the original force fields. [46,86]

	AMBER-DYE FF [46]		CHARMM-DYE [86]		
Bonds	Atom Type	R	Atom Type	R	ΔR
R(1,2)	ceg,ceg	1.4510	CDC21,CR252	1.4500	0.001
R(2,3)	cfg,ceg	1.3379	CDC21,CDC21	1.1940	0.144
R(3,4)	cfg,cfg	1.4511	CDC22,CDC21	1.4500	0.001
R(4,5)	c2g,cfg	1.3390	C2520,CDC22	1.3400	-0.001

2 Quantum Ensemble: Wigner-Distribution

In Section 3.2, we discussed our findings on the spectra obtained from the quantum ensemble through Wigner distribution. Figure S7 shows the spectra generated using B3LYP, CAM-B3LYP, and ωB97XD either using the same set of geometries (same Wigner ensemble) (solid

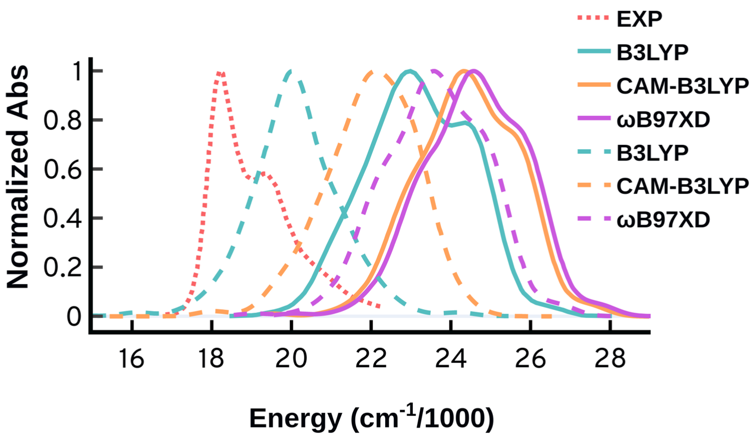

(a)

Figure S7: Normalized absorption spectra from an ensemble of 200 geometries generated using Wigner distribution and B3LYP, CAM-B3LYP, and ωB97XD on the same Wigner ensemble obtained from ωB97X/Def2SVP optimized structure (solid lines) and using different ensembles obtained from the equilibrium structure optimized using the respective methods and the Def2SVP basis set (dashed lines).

lines) or using different ensembles (dashed lines). The spectra depend profoundly by both the electronic structure method used to obtain the energies, as well as the method used to obtain the geometries. In all cases the spectra are significantly different from the experimental spectrum.

3 Franck-Condon Approaches

3.1 Probing the Herzberg-Teller effect

In polyatomic systems, weakly allowed and forbidden transitions can gain intensity from strongly allowed transitions via a vibronic coupling approximation. The Franck-Condon/Herzberg-Teller (FCHT) is a general approach where the zeroth and first order terms of the Taylor expansion are computed. If the transition dipole magnitude of the allowed transition is not large, the first order terms of the Taylor expansion will have more weights and this can have an effect on the spectra. Here we compare the Franck-Condon calculations within the zeroth-

order approximation, FC, and the first-order limit, FCHT, using the time-dependent (TD) approach at 298K. Figure S8 shows that truncating the Taylor expansion at the zeroth-order limit is satisfactory, and there is no need to worry about the Herzberg-Teller effects.

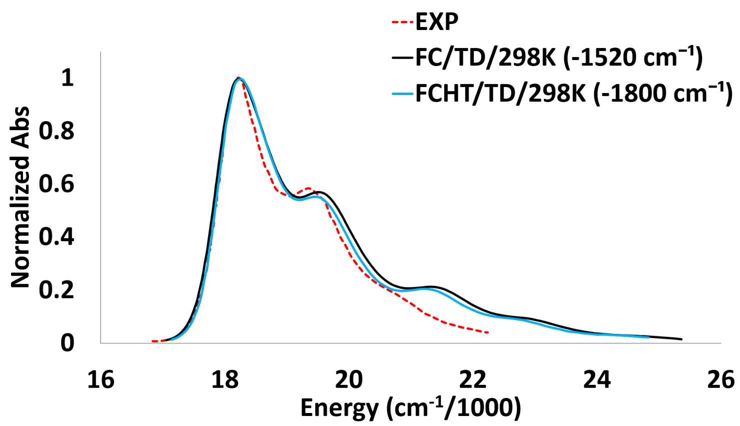

Figure S8: Comparing the spectra obtained from the Franck-Condon calculations within the zeroth-order approximation (FC) and the first-order limit (FCHT) using the time-dependent (TD) approach at 298K. The spectra are normalized and shifted to match the maximum of the experimental peak.

3.2 Huang-Rhys Factor

The magnitude of the Huang–Rhys factor depends on the displacement of the normal mode between the minimum on the ground state and the corresponding minimum on excited state, and is directly related to the Franck-Condon factors. This factor is of particular importance for symmetric modes where it can give quantitative information about shifts of nuclear equilibrium positions along the coordinates. Table S3 shows the Huang Rhys factors computed for the most important normal modes contributing to the spectrum. The 3rd normal mode is showing the largest displacement between the ground and first excited state.

3.3 Temperature effect

Here we show the stick spectrum obtained from FC/TI/AH/298K using ωB97XD/Def2SVP. Figure S9 shows that increasing the temperature lead to the population of the ground state

Table S3: Huang-Rhys Factors of the normal modes with the most prominent contribution to Cy3 vibronic spectrum.

Mode #	Huang-Rhys Factor
3	3.166
30	0.194
81	0.023
106	0.039
125	0.058

lower frequency modes, especially mode number 3, which contributes to the vibrational excitations along the spectrum bands.

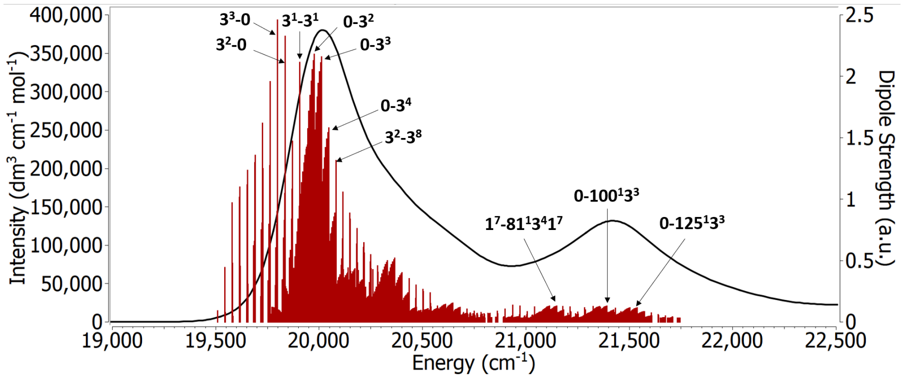

Figure S9: The vibronic spectrum obtained from structures optimized with ωB97XD/Def2SVP in gas phase at room temperature using the AH approach. The red sticks correspond to the vibrational excitations. The labels on the sticks are selected transitions.

4 Comparing the Ensemble and Franck-Condon Approaches

Similar to the comparisons done in Section 3.4, here we compare the ensemble and FC results using B3LYP and CAM-B3LYP. These comparisons demonstrate that our main conclusions hold for several functionals.

B3LYP: Figure S10 shows a comparison of the different approaches using B3LYP. Similarly, to what we show in the main paper for ωB97XD FC is the best approach. The inclusion of solvent improves the spectral shapes and energies of the spectrum obtained using the FC

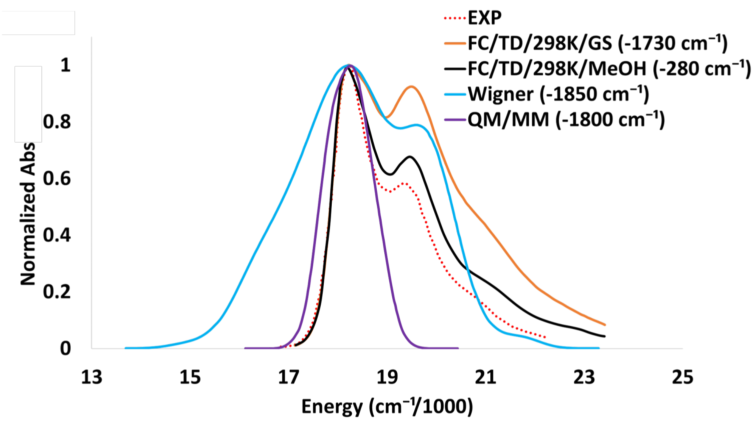

(a)

Figure S10: Comparing the spectra obtained from the Franck-Condon (FC), quantum sampling (Wigner), and classical sampling (QM/MM-MD) using B3LYP/Def2SVP. The spectra are normalized and shifted to match the maximum of the experimental peak.

approach compared to the one in the gas phase. Wigner distribution using initial conditions (geometries) from ωB97XD and energies from B3LYP is showing a spectrum that has three main spectral bands but it failed to reproduce the vibrational spacing, the relative intensities, and the exact width. Also, there is a shoulder to the right of the maximum. So, overall the spectrum is very different from experiment. Similarly, the classical ensemble and QM/MM calculations give a single symmetric peak and fail to capture any of the spectral signatures.

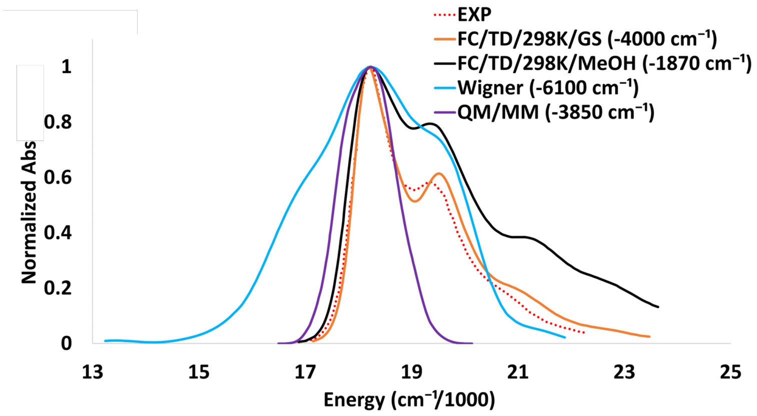

(a)

Figure S11: Comparing the spectra obtained from the Franck-Condon (FC), quantum sampling (Wigner), and classical sampling (QM/MM-MD) using CAM-B3LYP/Def2SVP. The spectra are normalized and shifted to match the maximum of the experimental peak.

CAM-B3LYP: Similar conclusions arise when using CAM-B3LYP. FC is again the best approach. Here however, unlike B3LYP, the inclusion of the solvent makes the spectrum worse, leading to incorrect relative band intensities. Wigner distribution produces a very wide spectrum that doesn't capture the spectral features. The QM/MM-MD CAM-B3LYP is similar to B3LYP with a single peak.

5 Structures of Cy3

Here we report the equilibrium structures of Cy3 in the ground and first excited state obtained from ωB97XD/Def2SVP optimizations using methanol as an implicit solvent via PCM, see Table S4.

Table S4: XYZ coordinates of Cy3 at the ground and first state (S_1) minimum obtained from ωB97XD/Def2SVP optimizations using methanol as an implicit solvent via PCM.

	Ground State			S_1		
	X	Y	Z	X	Y	Z
C	2.798949	-0.801099	0.023886	2.895582	0.851612	-0.022419
C	4.313308	-0.772789	0.017367	4.404113	0.745159	-0.015887
C	5.233219	-1.810103	0.040749	5.373321	1.734916	-0.036448
C	6.597211	-1.498097	0.029786	6.721220	1.359695	-0.025187
C	7.019211	-0.167572	-0.003804	7.082923	0.006370	0.006272
C	6.098751	0.884353	-0.028056	6.122329	-1.000904	0.027451
C	4.750021	0.549642	-0.017470	4.777513	-0.609262	0.015847
N	3.622239	1.397861	-0.037221	3.636812	-1.391706	0.030995
C	2.476751	0.696216	-0.012418	2.503794	-0.619621	0.010134
C	1.228423	1.322555	-0.012439	1.225412	-1.182904	0.012201
C	0.000003	0.664244	-0.000030	0.000000	-0.491509	-0.000006

C	-1.228419	1.322551	0.012360	-1.225413	-1.182905	-0.012214
C	-2.476747	0.696212	0.012376	-2.503794	-0.619621	-0.010143
C	-2.798951	-0.801103	-0.023922	-2.895582	0.851612	0.022420
C	-4.313309	-0.772788	-0.017355	-4.404113	0.745159	0.015893
C	-5.233224	-1.810099	-0.040712	-5.373321	1.734916	0.036464
C	-6.597215	-1.498089	-0.029713	-6.721220	1.359695	0.025206
C	-7.019210	-0.167563	0.003883	-7.082924	0.006370	-0.006261
C	-6.098747	0.884359	0.028109	-6.122329	-1.000904	-0.027450
C	-4.750018	0.549644	0.017491	-4.777513	-0.609262	-0.015848
N	-3.622233	1.397860	0.037222	-3.636812	-1.391705	-0.031005
C	-3.692127	2.844983	0.076274	-3.634040	-2.837068	-0.062587
H	-3.168181	3.228613	0.962744	-3.107797	-3.199885	-0.958010
H	-4.738161	3.159199	0.128227	-4.662382	-3.207984	-0.088142
H	-3.240074	3.276407	-0.828019	-3.137507	-3.240085	0.832793
H	-6.442970	1.918542	0.053244	-6.421215	-2.048994	-0.051427
H	-8.086570	0.061646	0.011316	-8.139620	-0.268146	-0.014450
H	-7.336798	-2.300588	-0.048101	-7.498143	2.126220	0.041194
H	-4.903577	-2.851240	-0.067567	-5.093285	2.790508	0.061161
C	-2.279743	-1.528372	1.229959	-2.407817	1.610996	-1.229447
H	-1.183049	-1.570269	1.258134	-1.312521	1.686901	-1.257009
H	-2.631484	-1.033093	2.145924	-2.745063	1.110493	-2.148011
H	-2.653938	-2.562004	1.230887	-2.815949	2.632204	-1.220623
C	-2.289075	-1.455870	-1.320829	-2.413473	1.545825	1.313843
H	-2.655214	-0.914408	-2.204517	-2.763640	1.004231	2.203871
H	-1.192270	-1.483920	-1.362858	-1.317686	1.608370	1.354830
H	-2.654527	-2.491194	-1.373966	-2.812366	2.569944	1.351051
H	-1.207249	2.413840	0.021066	-1.162546	-2.274342	-0.024138

H	0.000006	-0.424978	-0.000006	0.000000	0.596302	-0.000003
H	1.207250	2.413843	-0.021178	1.162546	-2.274342	0.024119
C	3.692137	2.844984	-0.076258	3.634040	-2.837068	0.062568
H	3.168391	3.228619	-0.962846	3.107796	-3.199890	0.957989
H	4.738182	3.159205	-0.127962	4.662382	-3.207985	0.088120
H	3.239879	3.276401	0.827935	3.137506	-3.240080	-0.832815
H	6.442978	1.918534	-0.053190	6.421215	-2.048995	0.051422
H	8.086571	0.061634	-0.011212	8.139620	-0.268147	0.014462
H	7.336791	-2.300598	0.048194	7.498143	2.126220	-0.041168
H	4.903568	-2.851243	0.067594	5.093285	2.790508	-0.061139
C	2.279775	-1.528370	-1.230007	2.407813	1.610989	1.229451
H	1.183082	-1.570260	-1.258217	1.312517	1.686896	1.257009
H	2.631549	-1.033098	-2.145964	2.745055	1.110481	2.148014
H	2.653962	-2.562005	-1.230918	2.815946	2.632198	1.220635
C	2.289026	-1.455857	1.320781	2.413478	1.545833	-1.313840
H	1.192219	-1.483884	1.362779	1.317691	1.608377	-1.354830
H	2.654456	-2.491188	1.373927	2.812371	2.569951	-1.351040
H	2.655152	-0.914401	2.204478	2.763648	1.004244	-2.203869
