# Supplementary figures and images for: Modeling the Electronic Absorption Spectra of the Indocarbocyanine Cy3
Source: Molecules. 2022 Jun 24;27(13):4062. doi: 10.3390/molecules27134062 (PMC9268038; doi:10.3390/molecules27134062)

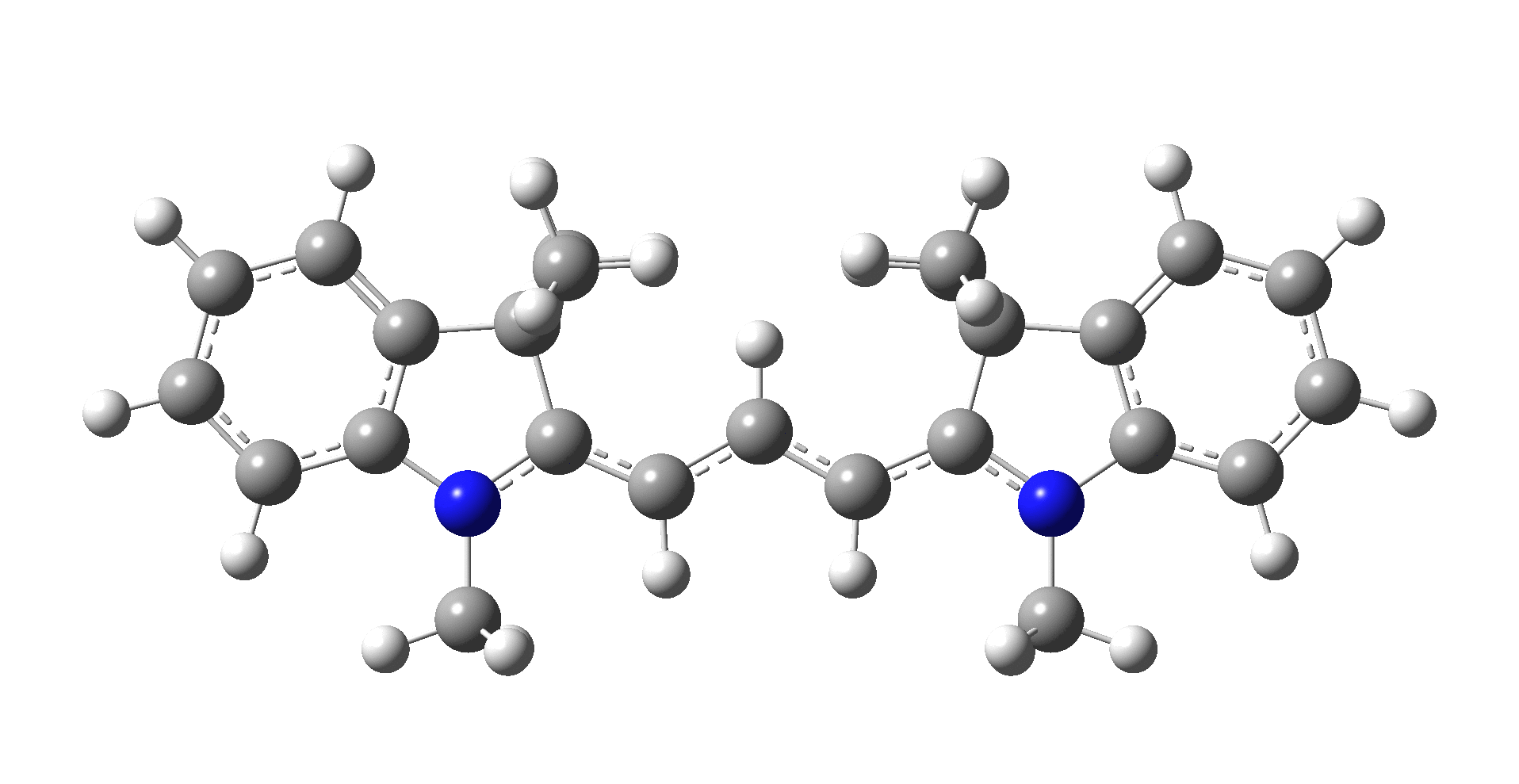

Supplement: Supplementary file 1 [file molecules-27-04062-s001.zip › normal-modes-movies/mode-100-movie.gif]

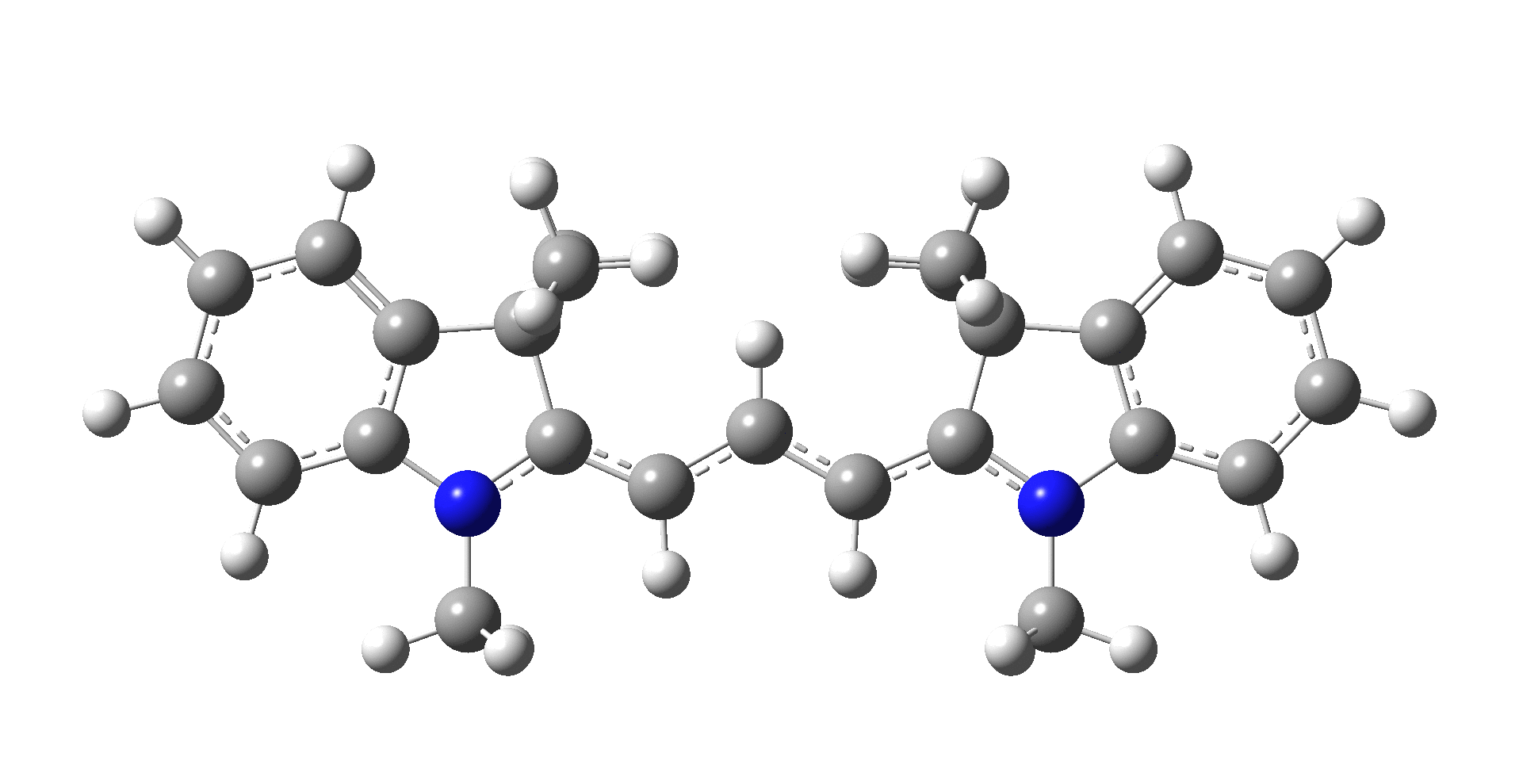

Supplement: Supplementary file 1 [file molecules-27-04062-s001.zip › normal-modes-movies/mode-106-movie.gif]

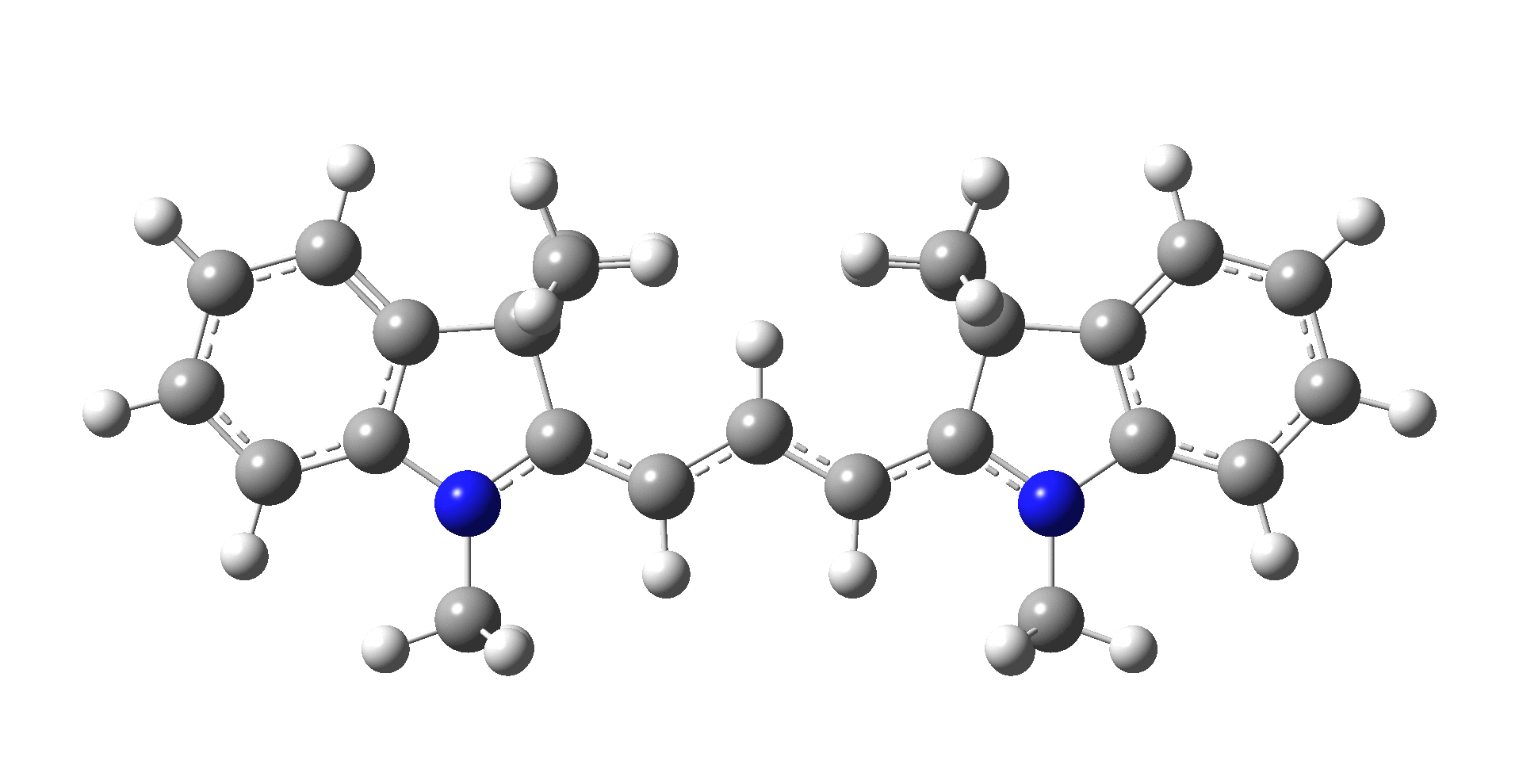

Supplement: Supplementary file 1 [file molecules-27-04062-s001.zip › normal-modes-movies/mode-125-movie.gif]

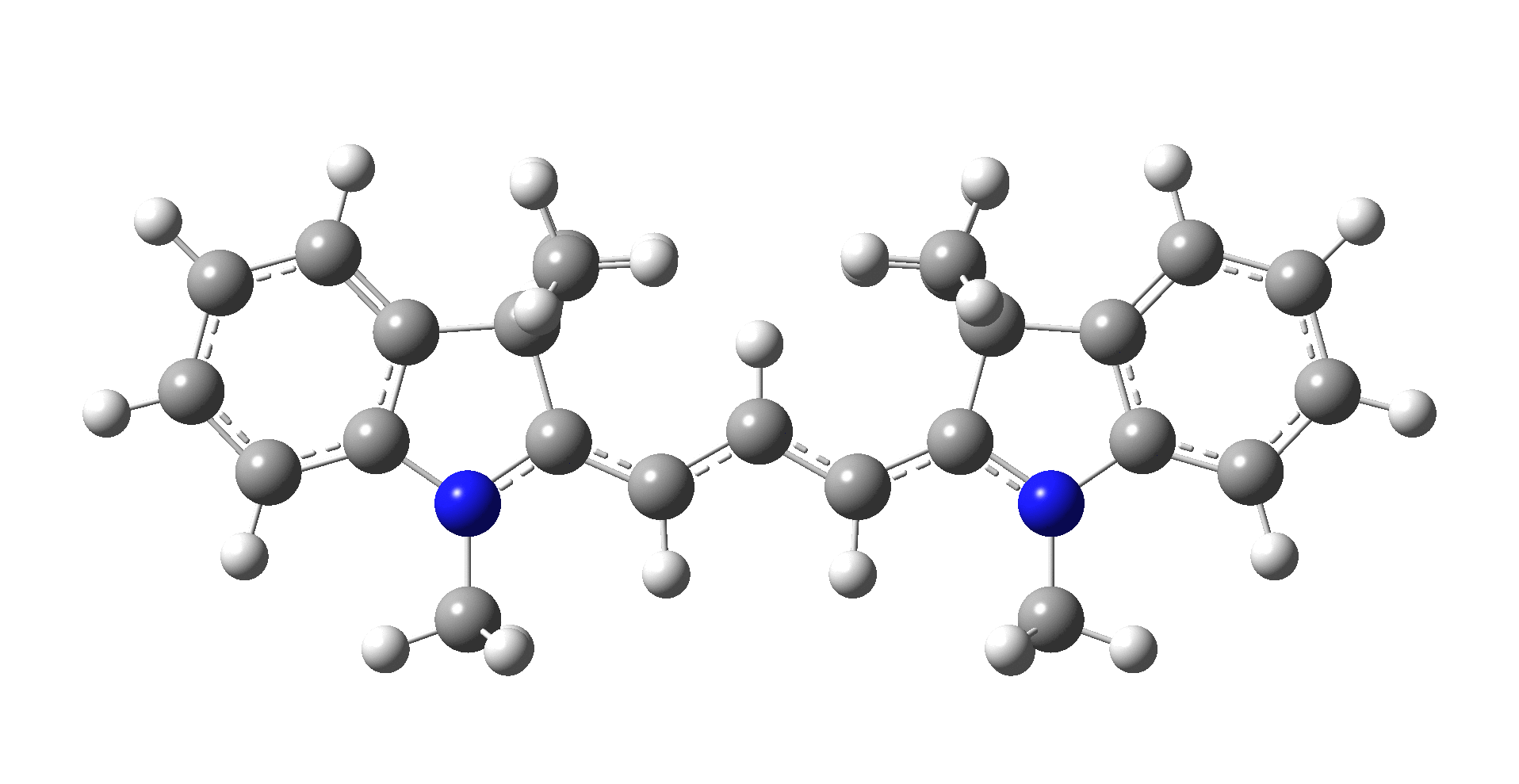

Supplement: Supplementary file 1 [file molecules-27-04062-s001.zip › normal-modes-movies/mode-3-movie.gif]

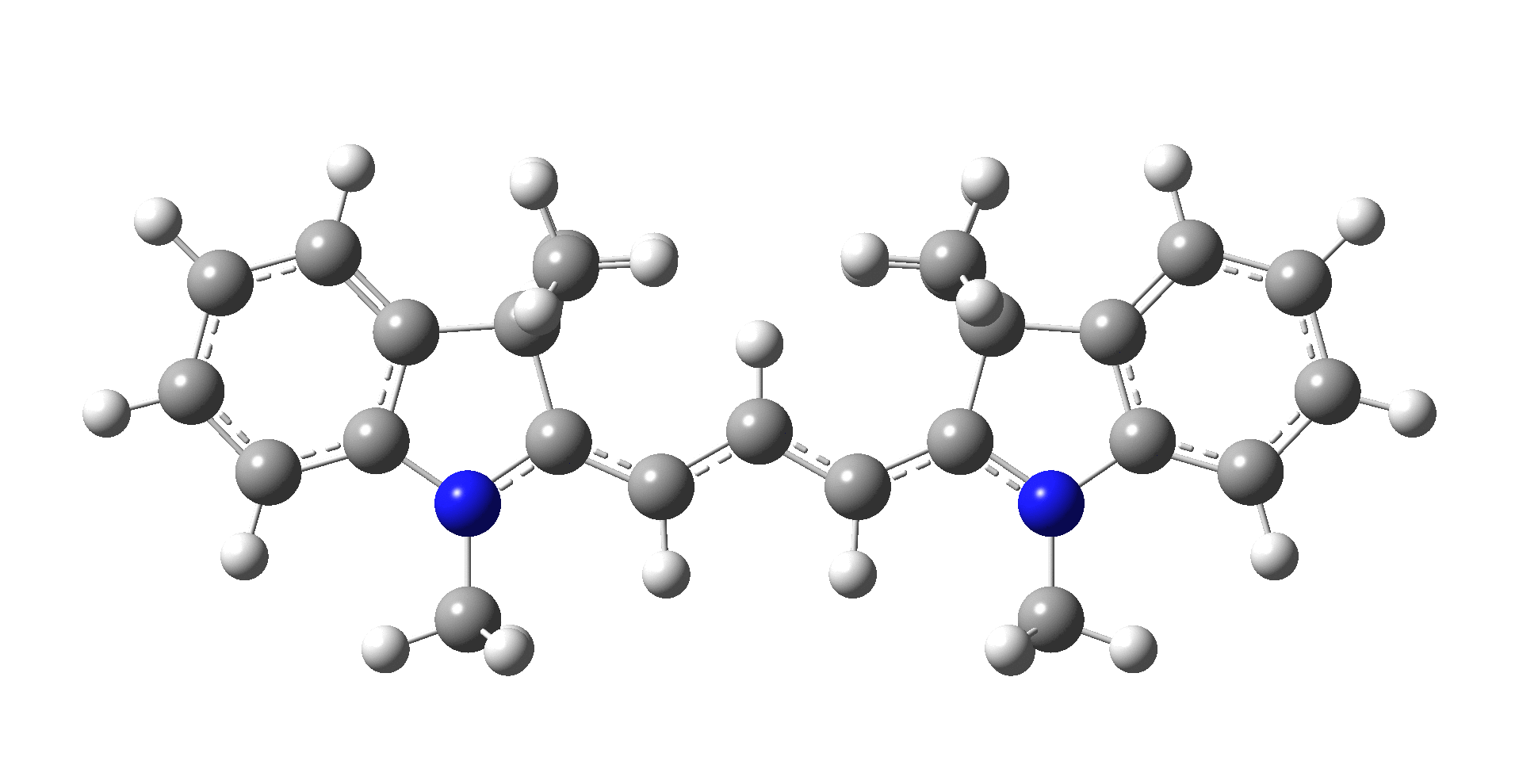

Supplement: Supplementary file 1 [file molecules-27-04062-s001.zip › normal-modes-movies/mode-30-movie.gif]

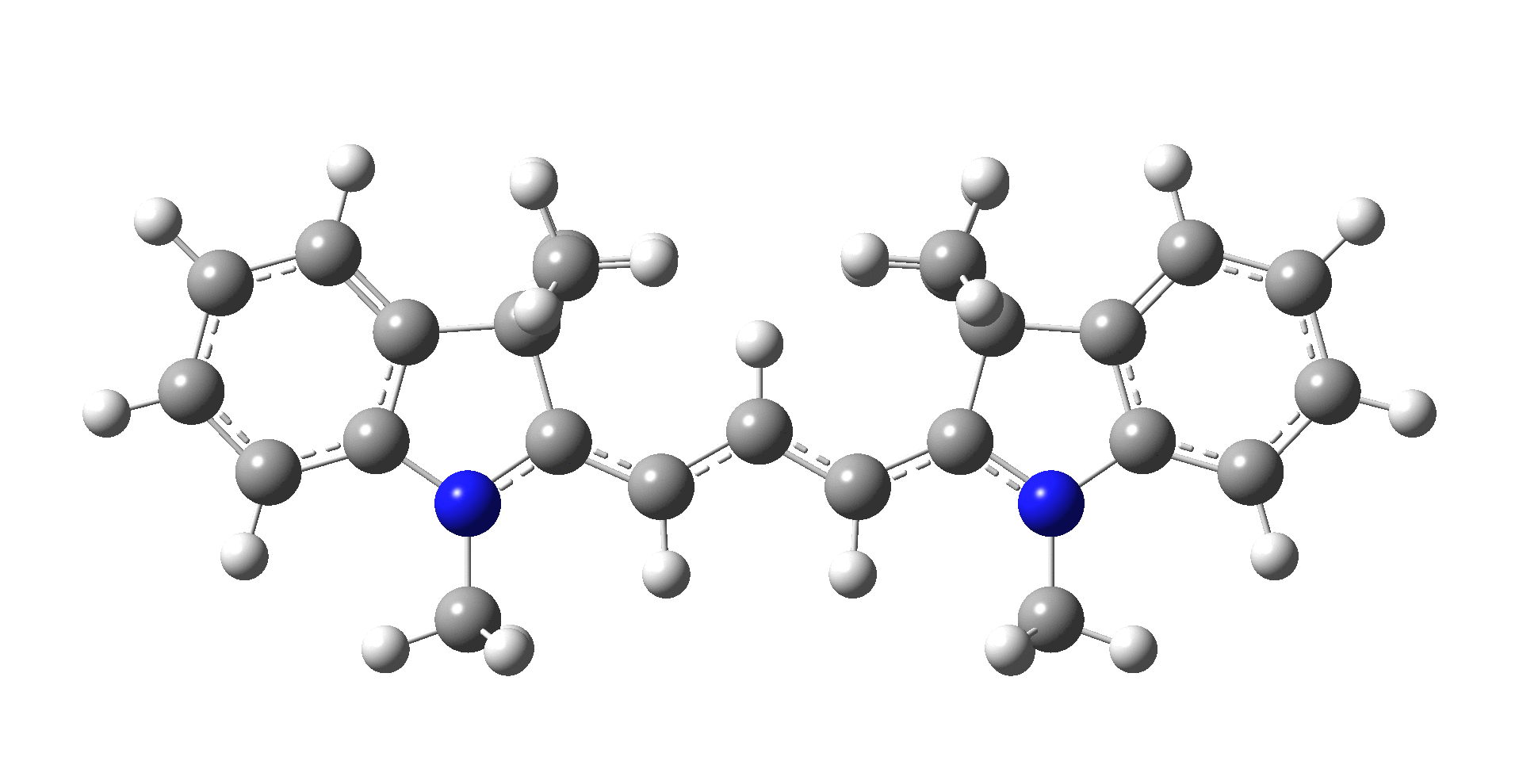

Supplement: Supplementary file 1 [file molecules-27-04062-s001.zip › normal-modes-movies/mode-81-movie.gif]
